# Paraganglioma of the Spermatic Cord: Case Report and Review of the Literature

**DOI:** 10.1100/tsw.2008.161

**Published:** 2008-12-25

**Authors:** G. Garaffa, A. Muneer, A. Abdel Raheem, A. Freeman, D. J. Ralph, S. Minhas, R. W. Rees

**Affiliations:** Department of Andrology, The Institute of Urology, University College London Hospitals, London, United Kingdom; ^2^ Department of Histopathology, University College London Hospitals, London, United Kingdom

**Keywords:** paraganglioma, phaeochromocytoma, cord, adrenal gland

## Abstract

Paragangliomas rarely involve the genitourinary tract. We present a case of a paraganglioma arising from the spermatic cord and review the literature on the topic.

## CASE REPORT

A 69-year-old man presented with unexplained weight loss and malaise. He had undergone a left upper lobectomy for a non–small cell carcinoma of the lung 5 years previously. Fluorodeoxyglucose Positron Emission Tomography (FDG PET) was performed and identified an avid lesion in the right spermatic cord (Fig. 1).

Physical examination confirmed the presence of a 2-cm mobile mass above the right testicle. An ultrasound scan confirmed a highly vascular lesion in the right spermatic cord. His testicular tumour markers and biochemical profile were all normal. He underwent an uncomplicated radical orchidectomy. Examination of the pathological specimen showed a well-circumscribed encapsulated mass in the spermatic cord, separate from the testis and epididymis. This was composed of nests of cells with moderate nuclear pleomorphism and abundant granular eosinophilic cytoplasm, with an intervening rich vascular network. There was no mitotic activity, necrosis, or lymphovascular invasion. Immunocytochemistry showed that the cells were positive for CD56, chromogranin, and synaptohysin, with an intermittent S100 positive sustentacular cell layer. In the absence of a lesion in the adrenals, a diagnosis of a primary paraganglioma of the spermatic cord was made (Fig. 2).

## DISCUSSION

Benign and malignant neoplasms of the spermatic cord are rare[1]. Lipomas, adenomatoid tumours, angiomyxomas, papillary cystoadenomas, and leiomyomas are the most frequently occurring benign neoplasms, while sarcomas and metastases represent the most frequent malignant counterparts[2,3].

Paragangliomas are catecholamine-secreting tumours originating from neuroendocrine cells of the paraganglia of the autonomic nervous system. These cells are characterized by numerous intracytoplasmic neurosecretory granules containing catecholamines or their precursors. They are located in the retroperitoneum

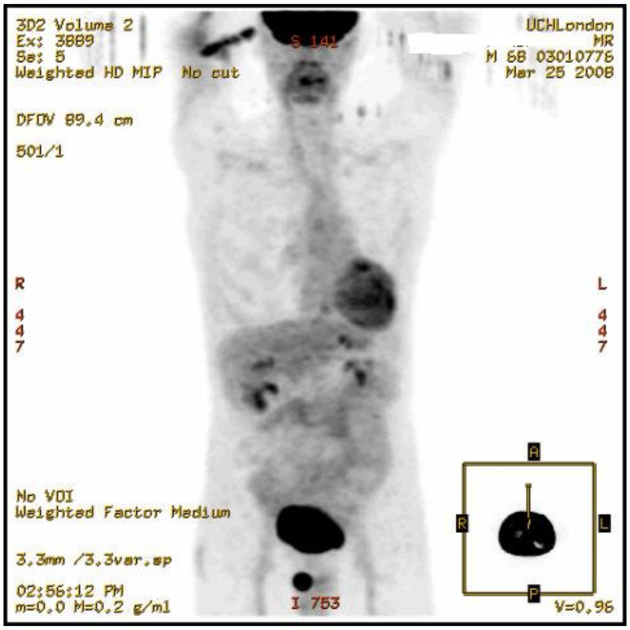

**FIGURE 1**. FDG PET shows an avid lesion in the right cord and rules out the presence of involvement of the adrenal glands.

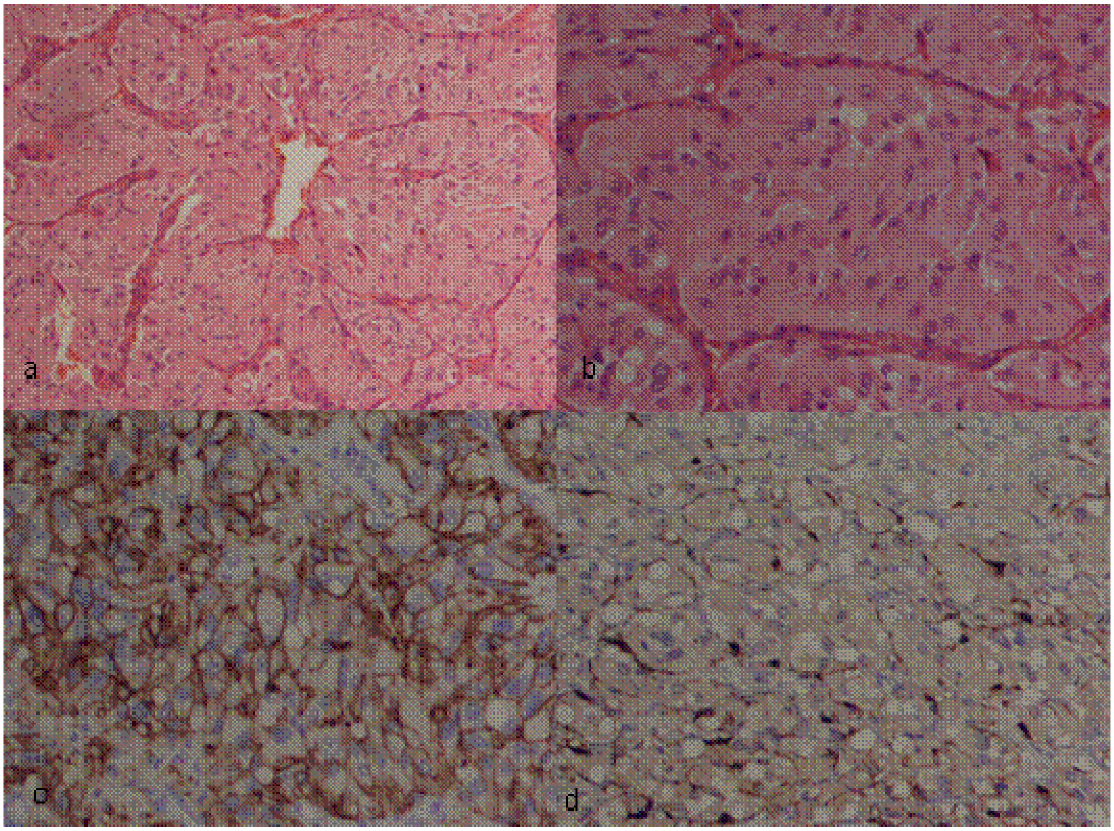

**FIGURE 2**. (a) A low-power view of the mass showing nested architecture and prominent vascularity (H&E, ×100 magnification). (b) Cells show moderate nuclear pleomorphism and abundant granular eosinophilic cytoplasm (H&E, ×200 magnification). (c) Strong CD56 membrane positivity (×100 magnification). (d) Scattered S100-positive sustentacular cells (×100 magnification)

along the para-aortic axis[4,5,6,7] and are similar to the neuroendocrine cells forming the adrenal medulla. Adrenal remnants have also been reported along the spermatic cord[8]. The microscopic features of tumours derived from these cells are similar, regardless of the location. In the adrenal medulla, the tumours are called phaeochromocytomas, whereas at extra-adrenal sites, they are termed paragangliomas[9,10]. These tumours are benign in around 90% of cases; the presence of distant metastases is the only reliable criterion to identify malignant cases.

Paragangliomas have been reported in unusual locations, such as the small intestine, gall bladder, lung, and thyroid[11]. Paragangliomas of the urinary bladder are not uncommon and are frequently associated with episodes of hypertension during voiding due to the release to catecholamines[11]. Very few cases involving the spermatic cords previously have been reported[5,7,9].

To our knowledge this is the first case in which an isolated paraganglioma of the spermatic cord has been identified on FDG PET scanning. The absence of haemodynamic symptoms in this patient is probably related to the small size of the tumour and its consequent inability to secrete a sufficient quantity of catecholamines.
